# Quantum Computing in a Diagnostic-First Quantum Residual Boosting Framework for Clinical Survival Analysis in Oncology and Cardiology

**DOI:** 10.3390/jcm15145387

**Published:** 2026-07-09

**Authors:** Cemil Colak, Burak Yagin, Gokhan Zorlu, Fahaid Al-Hashem, Sarah A. Alzakari, Amal K. Alkhalifa, Mohammadreza Aghaei

**Affiliations:** 1 Department of Biostatistics and Medical Informatics, Faculty of Medicine, Inonu University, Malatya 44280, Türkiye; cemil.colak@inonu.edu.tr; 2 Department of Biophysics, Faculty of Medicine, Inonu University, Malatya 44280, Türkiye; 3 Department of Physiology, College of Medicine, King Khalid University, Abha 61421, Saudi Arabia; 4 Department of Computer Sciences, College of Computer and Information Sciences, Princess Nourah Bint Abdulrahman University, P.O. Box 84428, Riyadh 11671, Saudi Arabia; 5 Department of Ocean Operations and Civil Engineering, Norwegian University of Science and Technology (NTNU), 7034 Alesund, Norway; 6 Department of Sustainable Systems Engineering (INATECH), Albert Ludwigs University of Freiburg, 79085 Freiburg, Germany

**Keywords:** quantum machine learning, survival analysis, kernel-target alignment, variational quantum circuits, hybrid quantum–classical modelling, concordance index, clinical prediction modelling

## Abstract

**Objective**: Survival prediction in oncology and cardiology requires models that can capture nonlinear prognostic structure while remaining interpretable, calibrated, and clinically safe. This study develops and evaluates a diagnostic-first hybrid quantum–classical framework for right-censored survival analysis. **Methods**: We introduce KTA-Survival (Kernel-Target Alignment for survival), a pre-training feasibility diagnostic that adapts kernel-target alignment to censored outcomes by comparing a quantum fidelity kernel with a concordance-based survival target kernel. We then propose QResid-Boost (Quantum Residual Boosting), a Cox-LASSO–anchored residual framework in which a variational quantum circuit is trained on martingale residuals through a Quantum-Skip-Residual architecture. A sigmoid-bounded scalar gate, α, constrains the quantum contribution and allows the model to reduce to the classical baseline when the residual signal is uninformative. The framework was evaluated on GBSG2 (German Breast Cancer Study Group 2; *n* = 686), FLChain (serum free light chain; *n* = 1500), WHAS500 (Worcester Heart Attack Study; *n* = 500), and a synthetic Weibull positive-control dataset containing high-frequency periodic interactions. **Results**: On the GBSG2 hold-out partition, Random Survival Forest achieved the highest concordance (C = 0.7188), followed by the Stacking ensemble (C = 0.7128), Cox-LASSO (C = 0.7019), and QResid-Boost (C = 0.7016). The leading classical and hybrid models did not differ significantly by paired bootstrap testing, whereas all outperformed the pure quantum variants. In the synthetic positive-control cohort, QResid-Boost improved over Cox-LASSO by ΔC = +0.0397, demonstrating that the quantum residual can add value when nonlinear periodic structure remains after the linear baseline. KTA-Survival yielded positive ΔKTA values across the evaluated datasets and correctly identified the regime in which the quantum residual produced its largest measurable gain. **Conclusions**: The proposed diagnostic-first framework reframes quantum survival modelling as a gated enrichment strategy rather than an unconstrained replacement for classical risk models. In low-dimensional clinical cohorts where linear structure already explains most prognostic signal, the framework behaves conservatively; when residual nonlinear structure is present, it can provide measurable improvement without uncontrolled model drift.

## 1. Introduction

Reliable prediction of time-to-event outcomes is central to precision medicine in oncology, cardiology, and other clinical domains in which censored follow-up is the norm. Cox proportional hazards modelling [1] remains a foundational method because it produces interpretable hazard ratios and performs well under modest sample sizes. Its principal limitation is structural: the log-hazard is assumed to be a linear combination of covariates unless nonlinearities are manually engineered. Consequently, threshold effects, high-frequency dependencies, and complex marker interactions may remain unmodelled. Machine-learning approaches such as Random Survival Forests [2] and DeepSurv [3] relax this restriction and have become important benchmark methods, but their gains are often accompanied by reduced transparency and additional calibration concerns. Traditional survival prediction therefore spans a spectrum: semi-parametric Cox regression offers interpretable, well-calibrated hazard ratios but presupposes log-linear and proportional-hazards structure; penalised Cox variants such as Cox-LASSO add automatic variable selection but retain the linear functional form; and fully non-parametric or deep learners (Random Survival Forests, DeepSurv, etc.) capture nonlinearities at the cost of interpretability and calibration stability. The framework proposed here differs from all of these in that it does not replace the classical risk model. Instead, it retains a Cox-LASSO backbone as the primary predictor and confines any nonlinear (quantum) capacity to a bounded, residual-only correction that is activated only after a pre-training feasibility screen indicates that exploitable nonlinear structure remains. This positions the contribution as a gated enrichment of established survival methods rather than as a competing standalone model.

These methodological tensions have direct clinical relevance. Established risk tools in oncology and cardiology, including the PREDICT breast cancer prognostic model [4,5], the Oncotype DX Recurrence Score in hormone-receptor-positive breast cancer [6], and the GRACE 2.0 post-infarction mortality score [7], were developed around specific clinical feature sets and conventional modelling assumptions. Although these instruments are clinically valuable, they do not systematically test whether nonlinear feature interactions contain additional prognostic information. Overestimation of recurrence risk can expose patients to unnecessary treatment burden, whereas underestimation can delay or prevent appropriate escalation. A clinically credible enrichment model should therefore satisfy two conditions: it should be screened for likely incremental value before deployment, and it should default to an interpretable classical baseline when the added modelling capacity is not warranted [8,9,10].

Quantum machine learning has been proposed as one route to this kind of nonlinear enrichment. Variational quantum circuits (VQCs) map input variables into high-dimensional Hilbert spaces through parameterised feature maps, allowing interactions that are nonlinear in the observed feature space to become linearly accessible after embedding [11,12]. Quantum kernels based on circuit fidelities have been associated with formal separation results in supervised learning under specific assumptions [13]. At the same time, data encoding and re-uploading strategies shape the expressive class of VQC models and determine whether a circuit is plausibly aligned with the structure of the prediction task [14,15].

Despite this theoretical appeal, the empirical performance of quantum models on small tabular clinical datasets has been inconsistent. Randomly initialised variational circuits can suffer from barren plateaus, where gradients vanish and optimisation becomes unstable [16,17]. Moreover, for low-dimensional clinical feature sets, a quantum embedding may be only marginally different from a well-tuned classical kernel, which limits the prospect of measurable improvement in concordance [18]. These limitations are amplified by the noise budget of present-day noisy intermediate-scale quantum [19]. The central question is therefore not whether a quantum model can be made more expressive, but whether one can identify, before training, the data regimes in which quantum capacity is likely to contribute useful residual information.

This study addresses that question through a diagnostic-first survival-modelling framework. First, KTA-Survival adapts kernel-target alignment [20] to right-censored outcomes by replacing the binary class kernel with a concordance-based survival target kernel. This makes it possible to compare the geometry of an untrained quantum embedding with the survival ranking before any costly optimisation is attempted. Second, QResid-Boost combines a Cox-LASSO baseline [21] with a quantum learner trained on martingale residuals [22], thereby restricting the quantum branch to the signal not already captured by the classical model. Third, the Quantum-Skip-Residual circuit implements a bounded additive correction inspired by residual learning [23]; the scalar gate α controls how far the final risk score may depart from the classical baseline. To make the explanation supporting these objectives explicit, each is motivated by a specific limitation identified above. The feasibility-screening objective (KTA-Survival) addresses the risk that, on small and low-dimensional clinical cohorts, a quantum embedding may differ only marginally from a well-tuned classical kernel; screening the geometric alignment between the untrained embedding and the survival target before any optimisation identifies, in advance, the regimes in which quantum capacity is worth training. The residual-confinement objective (QResid-Boost) addresses the interpretability and calibration costs of unconstrained machine-learning survival models; by training the quantum learner only on the martingale residuals of a fitted Cox-LASSO baseline, the interpretable classical model remains the primary predictor and the quantum branch can act only on signal the baseline has not captured. The bounded-correction objective (the sigmoid gate α) addresses the clinical-safety requirement that an enrichment model should not drift uncontrollably from an established baseline; constraining the quantum contribution to a learnable, bounded fraction guarantees that the framework reduces to the classical model when no residual structure is present. These three objectives therefore operationalise, point by point, the diagnostic-first principle that motivates the study. The methodological innovation of the framework is therefore not the use of any single existing technique, but the integration of three coupled ideas that, to our knowledge, have not previously been combined for censored survival data: (i) extending kernel-target alignment from the binary-classification setting to a concordance-based survival target kernel, yielding a training-free feasibility diagnostic for quantum embeddings under right-censoring; (ii) restricting the quantum learner to martingale residuals of a fitted Cox-LASSO model, so the quantum branch can only explain prognostic signal that the classical model has demonstrably failed to capture; and (iii) bounding the quantum contribution through a learnable sigmoid gate that guarantees graceful degradation to the classical baseline. The theoretical contribution is thus the formulation of quantum survival modelling as a screened, residual, and bounded enrichment problem rather than an unconstrained estimation problem.

We evaluate the framework on three public clinical survival cohorts and one synthetic positive-control dataset. The real-data analysis uses the German Breast Cancer Study Group 2 cohort [24], the Worcester Heart Attack Study (WHAS500) curated in applied survival-analysis work [25], and the FLChain serum free-light-chain dataset [26]. The synthetic Weibull dataset contains explicitly periodic feature interactions and is included to test whether the quantum residual can capture structure unavailable to a linear Cox-LASSO baseline. Classical and hybrid models are compared against Random Survival Forests, Cox-LASSO, DeepSurv, and an SLSQP-optimised Stacking ensemble [27]. Rather than positioning quantum modelling as a universal competitor to established methods, the study asks when, how, and under what safeguards a quantum residual component should be deployed.

The article proceeds as follows. Section 2 describes the datasets, preprocessing pipeline, variational quantum circuit, KTA-Survival diagnostic, QResid-Boost architecture, Stacking ensemble, NISQ simulation protocol, and statistical evaluation. Section 3 reports cohort characteristics, internal benchmarks, ablation analyses, external validation, multi-dataset diagnostic behaviour, NISQ robustness, and feature importance. Section 4 interprets these findings in relation to clinical prediction modelling, hybrid quantum–classical design, and the evidentiary requirements for clinical decision-support systems.

## 2. Materials and Methods

This section describes the cohorts, preprocessing strategy, model architectures, training protocol, and evaluation procedures. The implementation used Python 3.13 with PennyLane v0.40.0 [28], PyTorch v2.10.0, scikit-survival v0.23.1, lifelines v0.30.3, Optuna v4.9.0 [29], and scikit-learn v1.9.0. Experiments were run on an Ubuntu 22.04 LTS workstation equipped with an Intel Core i9-14900 CPU, 128 GB RAM, and an NVIDIA RTX 6000 Ada GPU with 48 GB VRAM (NVIDIA Corporation, Santa Clara, CA, USA). The complete end-to-end experiment, including hyperparameter optimisation and external validation, was completed in under two hours. This efficiency arises from several deliberate design choices rather than from large-scale parallelism. First, the quantum circuits were executed on a GPU-accelerated state-vector simulator (PennyLane lightning backend), so the six-qubit register required only a 2^6^ = 64-dimensional state vector that fits entirely in fast memory. Second, the modest circuit dimensions (six qubits, five layers) and the small, low-dimensional clinical cohorts keep both the forward simulation and the parameter-shift gradients inexpensive. Third, the diagnostic-first design avoids exhaustive quantum optimisation: KTA-Survival is computed in closed form from a single forward pass of the untrained circuit, and QResid-Boost trains the quantum branch only on Cox-LASSO residuals with early stopping rather than re-fitting a full survival model. Fourth, hyperparameter search was bounded (a fixed number of Optuna trials with pruning), and the classical baselines (Cox-LASSO, Random Survival Forest) are themselves computationally light on cohorts of this size.

### 2.1. Cohorts

Three real cohorts and one synthetic cohort were analysed. GBSG2 includes 686 patients with node-positive breast cancer, six routinely available prognostic features, and recurrence-free survival as the endpoint; the original trial is described by [24]. WHAS500 includes 500 patients hospitalised for acute myocardial infarction and was used as an external validation cohort with all-cause mortality as the endpoint [25]. FLChain comprises 1500 randomly subsampled participants from a population study of serum free light chains and mortality [26]. The synthetic Weibull cohort included 600 individuals and was generated with a hazard function containing one linear term and two high-frequency periodic interactions, so that Cox-LASSO could recover only part of the data-generating structure while a data re-uploading circuit could, in principle, capture the nonlinear residual pattern [15]. Because all real datasets were de-identified and publicly available, formal institutional review board approval was not required [30,31].

For completeness, the predictors, outcomes, event counts, and censoring rates of the three real cohorts are summarised here. GBSG2 contributed six routinely available clinicopathological predictors (age, menopausal status, hormonal-therapy status, number of positive lymph nodes, progesterone-receptor concentration, and oestrogen-receptor concentration), with recurrence-free survival as the endpoint, 686 patients, an event rate of 43.6% (299 events) and a corresponding censoring rate of 56.4%. WHAS500 contributed demographic, comorbidity, and acute-presentation predictors, with all-cause mortality as the endpoint, 500 patients, an event rate of 43.0% (215 events), and a censoring rate of 57.0%. FLChain contributed six predictors derived from serum free-light-chain measurements and routine demographics, with all-cause mortality as the endpoint, 1500 subsampled participants, an event rate of 26.9% (404 events) and a censoring rate of 73.1%. Missing values were handled exclusively by feature-wise median imputation fitted on the training partition (Section 2.2); no records were excluded because of missingness, and no predictor exceeded a negligible level of missingness in the analysed cohorts.

### 2.2. Preprocessing and Partitioning

Skewed variables, including positive-node count and hormone receptor concentrations, were transformed using log(1 + x), where x denotes the raw, untransformed non-negative value of the skewed predictor being transformed (the log(1 + x) form is used rather than log(x) so that genuine zero values, such as a positive-lymph-node count of zero, remain well defined). Missing values were imputed with feature-wise medians, and all predictors were standardised to unit variance. Each cohort was split into training, validation, and held-out test partitions using a 70%/15%/15% ratio stratified by event status. For the external validation experiment, the preprocessing pipeline fitted on the GBSG2 training partition was applied to WHAS500 without modification, thereby preserving a strict zero-shot transfer setting.

### 2.3. Variational Quantum Circuit

The variational quantum circuit used *N* = 6 qubits and L = 5 layers. The number of qubits was set equal to the dimensionality of the processed clinical feature vector (six predictors), so that each standardised covariate maps to one qubit under angle embedding without discarding or duplicating information at the encoding stage. The number of layers was chosen as a compromise between expressivity and trainability: deeper circuits increase representational capacity but also the risk of barren plateaus and of over-fitting on small cohorts, and preliminary experiments showed no consistent concordance gain beyond five layers at this width. These choices are examined directly in the ablation study, which varies ansatz type and ensemble size, and in Section 3.7, where widening the circuit to eight qubits and eight layers is shown to raise held-out concordance only for the highest-frequency synthetic target. Each layer applied *Y*-axis angle embedding with data re-uploading [14], followed by a strongly entangling rotation block [32]. Trainable parameters W ∈ ℝL × N × 3 were initialised from a Gaussian distribution with σ = 0.1 to reduce sensitivity to barren-plateau behaviour [16,17]. Six Pauli-Z expectation values were read out and passed through a compact classical head: a tanh-activated linear pre-encoder mapped the input vector to the qubit register, and a final linear layer converted the expectation vector into a scalar log-risk. The model was trained with a Cox partial-likelihood loss, mini-batches of 64 samples with at least two events per batch, Adam optimisation at a learning rate of 5 × 10^−3^, weight decay of 10^−4^, cosine annealing, unit-norm gradient clipping, and early stopping on validation concordance with patience of twelve epochs.

### 2.4. KTA-Survival Diagnostic

KTA-Survival measures the geometric compatibility between the quantum embedding and the survival target before model training. A survival target kernel KT ∈ ℝn × n was defined by setting KT(i, j) = 1 when a pair was observable and concordant, namely when (Ti < Tj ∧ δi = 1) or (Tj < Ti ∧ δj = 1), and KT(i, j) = 0; otherwise, diagonal entries were set to zero. The quantum kernel KQ was obtained from Pauli-Z expectations of the unoptimised circuit, with KQ(i, j) = exp(−‖zi − zj‖^2^/(2N)). A tuned classical RBF kernel KC served as the reference. In these expressions, n is the number of samples; KT, KQ and KC ∈ ℝ^(n × n) are the survival-target, quantum, and classical kernel matrices, respectively; Ti and δi are the observed time and event indicator of subject i; zi ∈ ℝ^N is the vector of N Pauli-Z expectation values read out from the unoptimised circuit for subject i; *N* = 6 is the number of qubits; ‖·‖ is the Euclidean norm; and ⟨·,·⟩F denotes the Frobenius inner product. To improve readability, these kernel and alignment definitions are rendered as displayed (separate-line) equations in the typeset version. Frobenius alignment between two centred kernels A and B was computed as A(A, B) = ⟨A, B⟩F/√(⟨A, A⟩F⟨B, B⟩F) [20]. We report KTAQ = A(KQ, KT) and ΔKTA = KTAQ − maxC A(KC, KT), where the subscript Q denotes the quantum kernel, C the classical reference kernel, and T the survival target kernel; A(·, ·) is the centred Frobenius kernel-target alignment defined above; and maxC denotes the maximum alignment taken over the family of candidate classical RBF kernels indexed by their length-scale (bandwidth) hyperparameter. In other words, ΔKTA contrasts the alignment of the quantum kernel with that of the best-aligned classical RBF kernel obtained over the tuned bandwidth grid. A positive ΔKTA indicates that the quantum embedding is more concordance-aligned than the best classical reference and justifies training the quantum residual branch.

### 2.5. QResid-Boost and Quantum-Skip-Residual

QResid-Boost begins with a regularised Cox-LASSO model [21]. After fitting the classical baseline, martingale residuals were computed as Mi = δi − Λ^0(Ti) exp(ηCoxi), where Λ^0 denotes the Breslow estimator of the cumulative baseline hazard [22]. A Quantum-Skip-Residual network, structurally identical to the VQC but initialised more conservatively with σ = 0.02, was then trained to regress these residuals under a weighted mean-squared-error objective. Event subjects were assigned weight 1.0 and censored subjects weight 0.8, reflecting the concentration of residual information among observed events. The final risk score was ηQ = ηCox + σ(αraw)VQC(x), where σ(z) = 1/(1 + e − z). Because σ(αraw) is bounded in (0, 1), the quantum branch cannot amplify risk without constraint; when the residual signal is weak, α approaches zero and the model reduces to Cox-LASSO. Quantum Kernel Alignment pre-training was optionally used to orient the circuit weights toward the KTA-Survival target before residual optimisation. Learning rate, α initialisation, and the censored-subject weight were tuned with Optuna [29]; the optimal censored weight for GBSG2 was 0.8. For notational consistency (as requested in review), a circumflex accent denotes a quantity estimated from the training data—for example, Λ^0 above is the Breslow estimate of the baseline cumulative hazard—while symbols without a circumflex, such as the observed time Ti and the event indicator δi, denote observed or fixed quantities; ηCox denotes the linear predictor of the fitted Cox-LASSO model. These accent and subscript conventions are applied consistently throughout the manuscript.

### 2.6. Stacking Ensemble

The Stacking ensemble [27] combined a three-member VQC ensemble, Random Survival Forest, Cox-LASSO, and DeepSurv. Component risk scores were min–max normalised on the validation partition. Non-negative stacking weights constrained to sum to one were estimated with sequential least-squares programming by maximising validation concordance. Once estimated, weights were frozen before evaluation on the internal held-out test set and the WHAS500 external validation cohort.

### 2.7. NISQ Simulation

NISQ behaviour was evaluated under three inference regimes: an ideal state-vector simulator, finite-shot sampling with 512 shots per circuit evaluation, and a noisy mixed-state simulation with depolarising noise (probability 0.003) plus bit-flip noise (probability 0.015) applied to each qubit at the end of each layer. The same trained weights were used in all three conditions, so the analysis isolated inference-time robustness rather than retraining effects.

### 2.8. Evaluation and Statistical Inference

This section specifies how predictive performance was quantified and how the uncertainty of those estimates was assessed. The objectives were threefold: to quantify discrimination (how well each model ranks subjects by risk), to quantify calibration (how well predicted risks match observed outcomes), and to attach formal uncertainty and significance statements to between-model comparisons, so that small numerical differences in the concordance index are interpreted in light of their sampling variability rather than at face value. The primary discrimination metric was Harrell’s concordance index [33], computed with inverse-probability-of-censoring weighting following [34]. Two-sided 95% confidence intervals were estimated using 200 stratified bootstrap resamples (B = 200), a count applied consistently to every confidence interval reported in the Results and Tables unless explicitly stated otherwise. Paired C-index differences were assessed with a bootstrap-based survival analogue of the DeLong procedure using 2000 resamples (B = 2000). Calibration was summarised with the Integrated Brier Score where applicable. Feature importance was estimated by permuting each predictor in the held-out test set and recording the resulting drop in concordance. Cox-LASSO regularisation was selected by five-fold cross-validated partial likelihood; Random Survival Forest parameters followed standard recommendations [2]; and DeepSurv hyperparameters were tuned over 30 Optuna trials using validation concordance as the objective.

### 2.9. Reporting-Guideline Alignment and Reproducibility Safeguards

The study was structured as a clinical prediction-model evaluation and is reported with explicit attention to TRIPOD+AI domains, including the modelling objective, source data, candidate predictors, outcome definition, partitioning strategy, validation design, discrimination, calibration, uncertainty, and intended clinical-use context [8]. This addition is important because the proposed framework is not presented as a deployable clinical decision-support system at this stage, but as an analytical validation of a diagnostically screened hybrid modelling strategy.

To support reproducibility, the manuscript now distinguishes between fixed design choices, tuned hyperparameters, and post hoc robustness analyses. The public availability of GBSG2, WHAS500, and FLChain, the explicit synthetic data-generating mechanism, fixed random seed, and complete reporting of benchmark variants provide a reproducible foundation. For a Q1-level submission (that is, a submission to a journal ranked in the first quartile, the top 25%, of its subject category by an indexing service such as Journal Citation Reports or Scopus CiteScore), the implementation should ultimately be archived with a permanent repository identifier, an executable environment file, and scripts that regenerate the tables and figures from raw inputs.

Clinical utility is deliberately separated from discrimination. Concordance, calibration, and bootstrap uncertainty establish analytical performance, whereas decision curve analysis and reclassification metrics are required to determine whether model output improves threshold-based clinical decision-making [10,35]. This separation prevents overstatement of modest C-index differences and aligns the interpretation with contemporary expectations for AI-enabled prediction models [8,9].

## 3. Results

The results are organised around seven questions: whether the cohorts provide clinically meaningful survival-prediction tasks; how the proposed models compare with established baselines; whether VQC ensembling and stacking add value; how QResid-Boost behaves when the residual signal is weak; which circuit-design choices are most influential; whether performance transfers across disease domains; and whether KTA-Survival anticipates the circumstances under which the quantum residual is useful.

### 3.1. Cohort Characteristics

**Figure 1:**
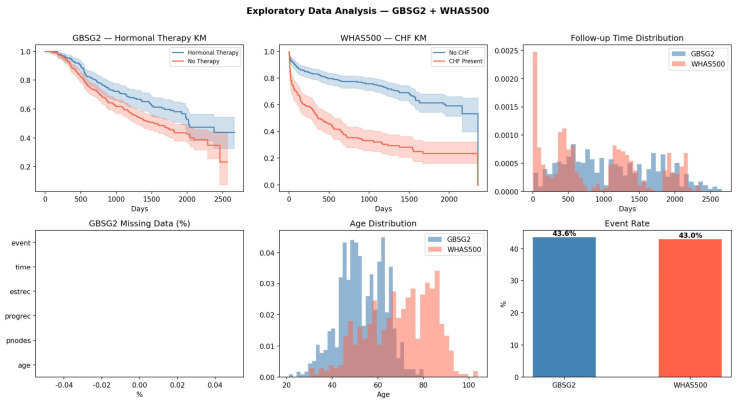
Exploratory analysis of GBSG2 and WHAS500. Top row: Kaplan–Meier curves stratified by hormonal therapy (GBSG2) and congestive heart failure (WHAS500); follow-up time distribution. Bottom row: missing-data quantification, age distribution, and observed event proportion.

Table 1 summarises the two principal cohorts. GBSG2 patients were substantially younger than WHAS500 patients (53.1 ± 10.1 years vs. 69.8 ± 14.5 years), reflecting the difference between breast-cancer recurrence and post-myocardial-infarction mortality. Event proportions were nevertheless similar (43.6% vs. 43.0%), allowing a meaningful assessment of discrimination under domain shift. Median follow-up was 1084 days for GBSG2 and 632 days for WHAS500. Figure 1 provides exploratory Kaplan–Meier curves, follow-up distributions, missing-data summaries, age distributions, and event-rate plots. These exploratory plots support three observations that frame the modelling task. First, the Kaplan–Meier panels show clear separation by the chosen stratifying variable in each cohort (hormonal therapy in GBSG2 and congestive heart failure in WHAS500), confirming that both cohorts carry genuine, clinically interpretable prognostic signal rather than near-random outcomes. Second, the follow-up and event-rate panels confirm adequate and comparable event proportions (43.6% versus 43.0%), so that the concordance index is estimated from a sufficient and comparable number of risk sets in both cohorts. Third, the missing-data panel indicates only low-level missingness that is fully resolved by the median-imputation step (Section 2.2), while the age-distribution panel makes explicit the marked demographic shift between the younger breast-cancer cohort and the older post-infarction cohort that later challenges external transfer (Section 3.6).

Clinically, GBSG2 represents a node-positive breast cancer cohort with variables that resemble the core elements of first-generation pathology-based prognostic tools, including the Nottingham Prognostic Index [36]. WHAS500, by contrast, represents post-acute myocardial infarction survival and contains demographic, comorbidity, and acute presentation variables broadly aligned with established cardiovascular risk scores such as TIMI [37] and GRACE 2.0 [7]. Therefore, the two cohorts provide complementary tests: internal discrimination in a cancer recurrence setting and external transfer to a clinically distinct cardiovascular endpoint.

### 3.2. Internal Benchmarks on GBSG2

Table 2 reports internal test performance on the GBSG2 hold-out partition. Random Survival Forest achieved the strongest single-model discrimination (C = 0.7188), followed by the Stacking ensemble (C = 0.7128), DeepSurv (C = 0.7035), Cox-LASSO (C = 0.7019), and QResid-Boost (C = 0.7016). The pure quantum models performed substantially worse: the single VQC reached C = 0.5564 under ideal simulation, C = 0.6191 with finite-shot sampling, and C = 0.5881 under the NISQ noise model; the three-member VQC ensemble reached C = 0.5062. These results indicate that the low-dimensional GBSG2 feature space is largely captured by classical survival models and that quantum capacity, when used without a classical anchor, does not recover the dominant prognostic signal. This conclusion is supported quantitatively by the magnitudes in Table 2. The pure quantum models clustered between C = 0.5062 (three-member VQC ensemble) and C = 0.6191 (512-shot VQC), that is, between chance level and weak discrimination, whereas every model that retained a classical anchor reached C ≈ 0.70–0.72; the paired bootstrap test confirmed that this classical-versus-quantum gap was statistically significant (*p* ≤ 0.033 for every classical-versus-quantum comparison, reaching *p* ≈ 0.001 for the widest gaps), in contrast to the non-significant differences among the classical and hybrid models. The feature-importance analysis provides a mechanistic explanation: a single predictor, the log-transformed positive-lymph-node count, accounted for a concordance drop of approximately 0.09 when permuted, indicating that most of the prognostic signal in GBSG2 is low-dimensional and near-linear and is therefore already captured by Cox-LASSO and Random Survival Forest, leaving little exploitable residual structure for an unanchored quantum embedding.

Paired bootstrap testing showed no statistically significant differences among RSF, Stacking, Cox-LASSO, and QResid-Boost at the 5% level (all paired *p*-values > 0.36; Table S1). In contrast, every classical or hybrid model significantly outperformed the pure quantum variants (paired bootstrap *p* ≤ 0.033 for every classical-versus-quantum comparison, reaching *p* ≈ 0.001 for the widest gaps; Table S1). Calibration analysis led to a similar conclusion: Cox proportional hazards modelling achieved an Integrated Brier Score of 0.1544, whereas QResid-Boost produced an IBS of 0.1924. Thus, although the quantum residual preserves much of the ranking behaviour of the baseline, its probability estimates would require additional recalibration before clinical use. The paired comparisons reported here are not separate from the tabulated results: they follow the bootstrap procedure defined in Section 2.8 (a survival analogue of the DeLong test with B = 2000 paired resamples) applied to the C-index point estimates and 95% confidence intervals already given in Table 2, whose pairwise overlap is what the test formalises. The absence of a significant difference among RSF, Stacking, Cox-LASSO, and QResid-Boost is the expected and intended outcome rather than a negative finding. Because GBSG2 is low-dimensional and its prognostic signal is largely linear (Section 3.9), the leading models occupy a narrow concordance band (C ≈ 0.70–0.72) with overlapping confidence intervals, so a cohort of this size is underpowered to resolve differences of the order of ΔC ≈ 0.003–0.017. For QResid-Boost in particular, statistical equivalence with Cox-LASSO is the designed behaviour of the bounded α gate: when no residual signal remains, the hybrid model is meant to collapse onto, and therefore not differ from, its classical baseline. The practical implication is that the quantum residual should be judged by its incremental calibration and net benefit, not by a concordance contest it is explicitly engineered not to win when the linear baseline already suffices.

Three points follow from Table 2. First, the leading models fall within a narrow concordance band, and their overlapping confidence intervals argue against over-interpreting small numerical differences in the C-index [34,38]. Second, the gap between the classical/hybrid models and the pure quantum models is large enough to be practically meaningful. Third, the calibration gap suggests that the present role of QResid-Boost is not to replace Cox-LASSO, but to provide a safety-gated residual extension whose clinical utility must be evaluated with calibration and decision-analytic measures in addition to discrimination.

**Figure 2:**
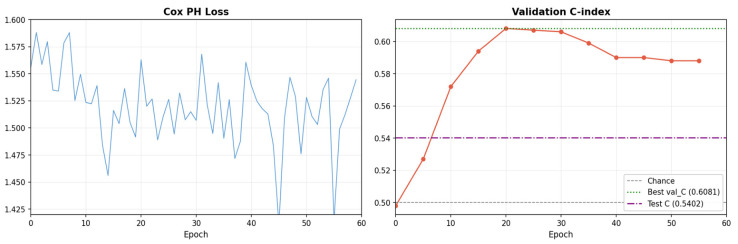
VQC-Survival training trajectory. (**Left**) Cox partial-likelihood loss across sixty epochs. (**Right**) validation concordance with chance, best-validation, and held-out test references annotated. In both panels, curve colors and line styles are as follows: the training loss is drawn in blue (solid), the validation C-index in red (solid with markers), and the reference lines denote the best validation C-index (green dotted), the test C-index (magenta dash-dot), and chance level (gray dashed).

**Figure 3:**
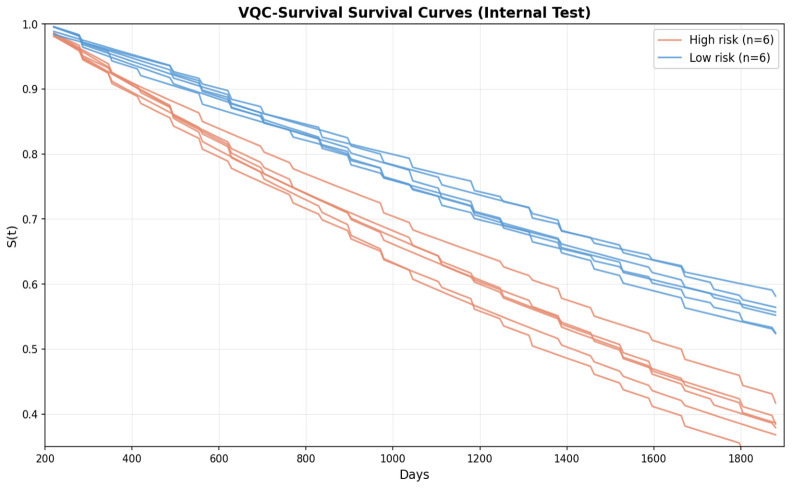
Predicted survival curves for the VQC-Survival model on the internal test partition. Six high-risk (red) and six low-risk (blue) subjects are stratified by the median model-predicted log-risk.

Figure 2 and Figure 3 illustrate, respectively, the optimisation behaviour and the practical output of the standalone VQC-Survival model and should be read together with the benchmark in Table 2. In Figure 2, the Cox partial-likelihood loss decreases smoothly over the sixty training epochs without divergence or oscillation, indicating that conservative initialisation and gradient clipping kept the circuit clear of barren-plateau instability; however, the validation-concordance trace plateaus only modestly above the chance reference and well below the held-out classical benchmark, which is the training-side manifestation of the weak standalone discrimination (C = 0.5564 under ideal simulation) reported in Table 2. Figure 3 translates this limited discrimination into clinical terms: although the model does separate the six designated high-risk subjects from the six low-risk subjects, the predicted survival trajectories overlap substantially, so the risk ordering produced by the unanchored quantum model is not sharp enough to support confident individual-level stratification. Taken together, the two figures reinforce the central argument of the study: a pure VQC trained on these low-dimensional clinical data optimises stably but does not, on its own, reach clinically useful discrimination, which is precisely the regime in which the diagnostic-first, residual-gated design is intended to keep the quantum branch inactive.

### 3.3. VQC Ensemble and Stacking Analysis

**Figure 4:**
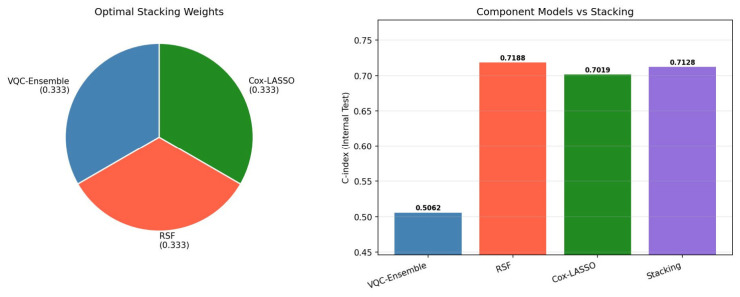
VQC ensemble member distribution. (**Left**) per-member validation concordance against the single-VQC ceiling. (**Right**) distribution of member concordances; the ensemble averaging combines variance from independent seeds.

**Figure 5:**
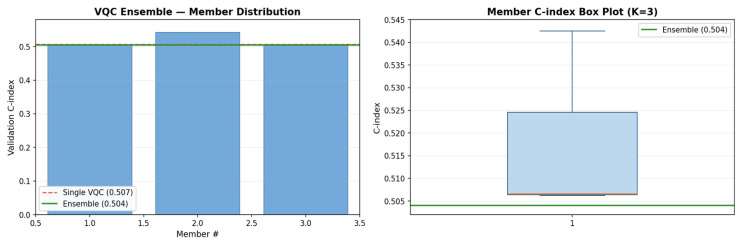
Optimal Stacking ensemble. (**Left**) SLSQP-optimal convex weights on validation, near-uniform across components (VQC-Ensemble, RSF, Cox-LASSO; ≈0.333 each). (**Right**) held-out internal C-index of each component versus the stacked ensemble. In the left panel, blue bars denote the validation C-index of each individual ensemble member (Member #), the red dashed line marks the single VQC baseline, and the blue solid line marks the ensemble score. In the right panel, the box plot summarizes the distribution of member C-indices for K = 3 (box: interquartile range; central line: median; whiskers: full data range), and the green solid line indicates the ensemble score.

Figure 4 shows that the three VQC ensemble members clustered around validation concordances of approximately 0.50–0.55, and the averaged ensemble did not improve over the best individual circuit. Figure 5 summarises the Stacking analysis. The SLSQP-optimised weights converged to an approximately uniform allocation across the retained components, and the final stacked C-index of 0.7128 remained below RSF alone. The validation-to-test difference was small (0.7177 vs. 0.7128), and five-fold cross-validation yielded a mean stacked Uno’s C of 0.7110 (SD = 0.008), suggesting that the ensemble result was stable but not superior to the strongest individual baseline.

### 3.4. QResid-Boost on GBSG2

**Figure 6:**
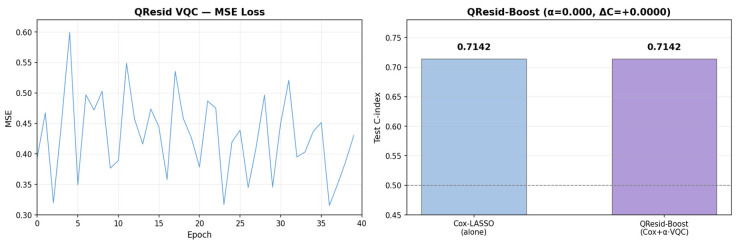
QResid-Boost dynamics on GBSG2. (**Left**) VQC residual mean-squared-error loss over forty epochs. (**Right**) held-out concordance for Cox-LASSO alone versus the QResid-Boost combination; α stabilises near 0.145 with a negligible ΔC (≈−0.0003 on the test partition).

Figure 6 summarises QResid-Boost training on GBSG2. The residual mean-squared-error loss fluctuated between approximately 0.32 and 0.55 over forty epochs, and the learned α gate stabilised near 0.145. Test concordance was virtually unchanged relative to Cox-LASSO (0.7016 vs. 0.7019; ΔC = −0.0003). This behaviour is clinically important: the framework did not force a quantum correction where little residual signal was available. Instead, the bounded gate kept the hybrid model close to the classical baseline, demonstrating the intended safety mechanism.

### 3.5. Ablation Study

**Figure 7:**
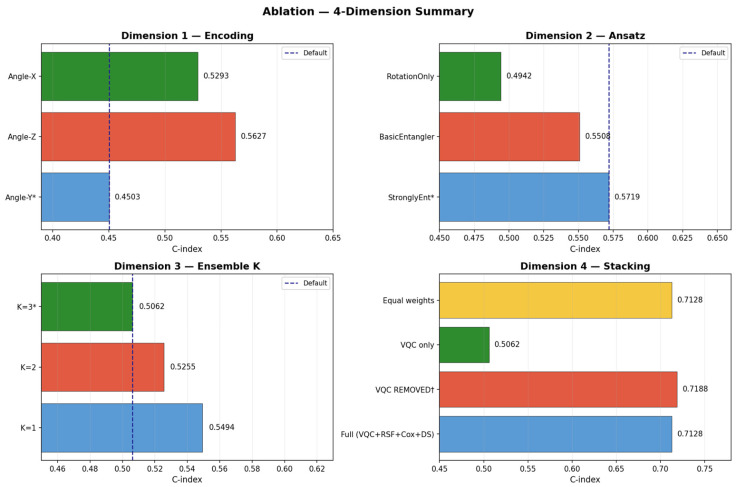
Four-dimensional ablation summary. Each subplot fixes three design choices at the default while sweeping one. Dashed line indicates the default configuration. An asterisk (*) marks the default (selected) configuration for each dimension, coinciding with the dashed reference line; the dagger (†) in Dimension 4 denotes the ablation configuration in which the VQC component was removed from the full ensemble.

Figure 7 and Table 3 report four ablation axes: embedding rotation, ansatz type, VQC ensemble size, and stacking composition. Encoding was the most influential circuit-level factor. Z-rotation and X-rotation outperformed the default Y-rotation in the standalone VQC setting, with C = 0.5627 and C = 0.5293 compared with C = 0.4503. Strongly entangling layers outperformed both basic entanglers and rotation-only circuits, supporting the need for an entanglement budget. Increasing the VQC ensemble size did not improve performance, and removing the VQC component from the Stacking ensemble raised concordance from 0.7128 to 0.7188.

The ablation results reinforce the diagnostic-first argument. The quantum component is sensitive to design choices, especially feature encoding, but sensitivity alone does not imply clinical usefulness. On GBSG2, the VQC contribution appears to add residual variance rather than orthogonal prognostic information. This interpretation is consistent with ensemble theory, where stacking improves performance only when component errors are sufficiently diverse and informative [39,40]. Future implementations should therefore consider validation-aware VQC pruning rather than uniform averaging across randomly seeded circuits.

### 3.6. External Validation on WHAS500

**Figure 8:**
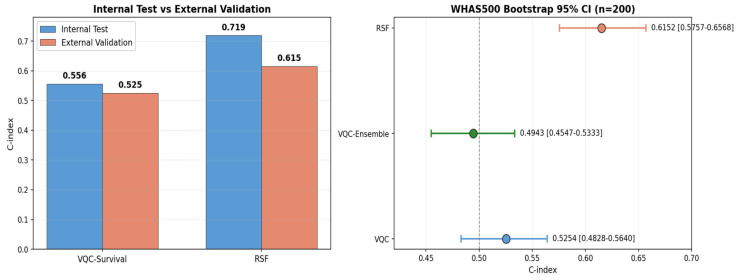
Internal versus external concordance. (**Left**) bar comparison for VQC-Survival and RSF on the internal hold-out partition and on the WHAS500 external cohort. (**Right**) WHAS500 bootstrap 95% confidence intervals (*n* = 200 resamples) for VQC, VQC-Ensemble, and RSF.

Table 4 and Figure 8 report zero-shot transfer from GBSG2 to WHAS500. All models showed reduced external discrimination, as expected given the shift from breast-cancer recurrence to post-infarction mortality. The Stacking ensemble achieved C = 0.6211, and RSF achieved C = 0.6152. The pure quantum models remained near chance, with C = 0.5254 for VQC-Survival and C = 0.4943 for the VQC ensemble. These findings indicate that neither classical nor quantum models should be transferred across clinically dissimilar survival tasks without local recalibration or retraining.

The smaller apparent decline observed for pure quantum models is not evidence of greater robustness; rather, their internal performance was already low, leaving less room for further deterioration. In absolute terms, RSF and Stacking retained clinically meaningful discrimination, whereas the confidence intervals for the quantum-only models approached chance. The external validation analysis therefore supports a modular deployment strategy: retrain the classical Cox-LASSO backbone on local cardiovascular data and use the quantum residual only if KTA-Survival indicates that additional nonlinear structure is present.

### 3.7. Multi-Dataset KTA-Survival Diagnosis

**Figure 9:**
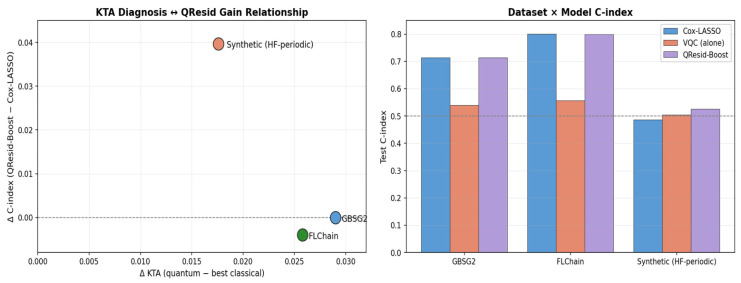
KTA-Survival diagnosis across datasets. (**Left**) relationship between ΔKTA (quantum minus best classical) and the QResid-Boost concordance gain over Cox-LASSO. The synthetic high-frequency cohort exhibits the largest ΔC (+0.04), while GBSG2 and FLChain exhibit ΔC ≈ 0—exactly the safety-gated behaviour expected when the linear baseline already exhausts the prognostic signal. (**Right**) per-dataset concordance for Cox-LASSO, VQC alone, and QResid-Boost.

Table 5 and Figure 9 evaluate KTA-Survival across GBSG2, FLChain, and the synthetic positive-control cohort. ΔKTA was positive for all three datasets: +0.0290 for GBSG2, +0.0241 for FLChain, and +0.0176 for the synthetic cohort. On GBSG2, QResid-Boost improved over Cox-LASSO in the multi-dataset evaluation split by ΔC = +0.0169; on FLChain, performance remained essentially unchanged (ΔC = −0.0015); and on the synthetic high-frequency cohort, QResid-Boost improved over Cox-LASSO by ΔC = +0.0397. The synthetic result is the strongest positive-control evidence that the quantum residual can add value when the data-generating process contains nonlinear periodic structure that a linear baseline cannot capture. In clinical terms, this synthetic cohort is a controlled stand-in for prognostic settings in which risk depends on non-monotonic or cyclical relationships rather than on simple linear effects, for example threshold and interaction effects between biomarkers, dose- or time-periodic exposures, or circadian and seasonal physiological signals. The result should therefore be read not as direct evidence of benefit in the three real cohorts studied here, where the prognostic signal was largely linear and the gate correctly remained near zero, but as a proof of principle that, when such nonlinear structure is genuinely present in clinical data, the diagnostic-first framework can detect it (positive ΔKTA) and exploit it (positive ΔC) instead of leaving it unmodelled. This delineates the clinical use case for which the method is intended and motivates its future evaluation on higher-dimensional omics, radiomics, or longitudinal cohorts where nonlinear interactions are more likely to occur.

KTA-Survival should be interpreted as a feasibility screen rather than a performance guarantee. A positive ΔKTA indicates that the untrained quantum embedding is geometrically compatible with the survival ranking, but the realised C-index still depends on sample size, residual signal strength, optimisation stability, readout calibration, and circuit capacity. The FLChain result illustrates this distinction: despite a positive ΔKTA and a high α value, QResid-Boost did not improve over an already strong Cox-LASSO baseline.

Bootstrap percentile intervals for ΔKTA were positive for GBSG2 [0.011, 0.048], FLChain [0.006, 0.042], and the synthetic dataset [0.001, 0.035]. Permutation testing with 1000 label reassignments yielded *p*-values of 0.003, 0.009, and 0.031, respectively, indicating that the observed alignment exceeded chance. In the synthetic cohort, the VQC still captured the periodic terms only partially: regression of the true log-relative hazard against the VQC-predicted log-risk yielded an overall R2 of 0.11, while the partial R2 for the linear component alone was 0.39. Increasing circuit width and depth to 8 qubits and 8 layers raised held-out Uno’s C to 0.55, suggesting that the default six-qubit, five-layer circuit was under-parameterised for the highest-frequency structure.

These findings clarify the operating logic of the diagnostic-first paradigm. ΔKTA and ΔC were directionally aligned, but not monotonically proportional, because realised performance depends jointly on kernel compatibility, the residual signal left by Cox-LASSO, and the capacity of the circuit [20,41]. The bounded α gate should likewise be interpreted as an indicator of residual engagement, not as a guarantee of discrimination gain. The synthetic positive control is therefore crucial: it demonstrates that QResid-Boost can improve performance when the residual structure was deliberately designed to favour periodic encodings, reducing the risk that the quantum benefit is a post hoc artefact [15].

### 3.8. NISQ Robustness

**Figure 10:**
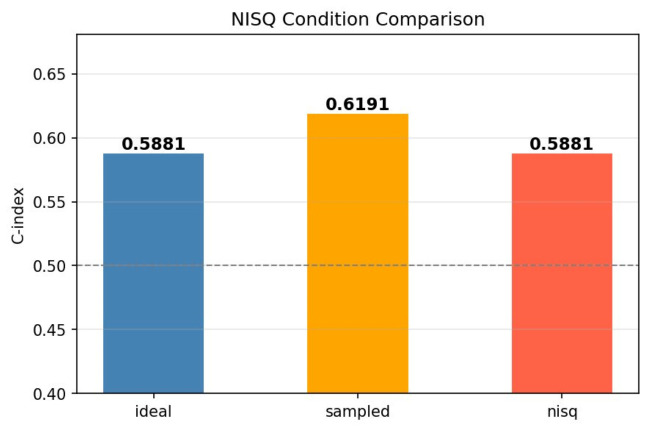
NISQ regime comparison. Held-out concordance under ideal state-vector simulation, 512-shot sampling, and depolarising-plus-bit-flip noise.

Figure 10 summarises the NISQ robustness analysis. Ideal simulation yielded C = 0.5564, finite-shot sampling with 512 shots yielded C = 0.6191, and depolarising-plus-bit-flip noise yielded C = 0.5881. Although the point estimate under noise slightly exceeded the ideal value, the two regimes’ confidence intervals overlapped substantially (ideal, [0.5152, 0.5970]; NISQ, [0.5471, 0.6286]), indicating that concordance was not materially degraded by noise. This robustness is plausible because concordance is rank-based: if noise contracts expectation-value magnitudes without substantially reordering predicted risks, discrimination remains stable. The finite-shot improvement should be treated cautiously and is most likely a variance-regularisation artefact rather than evidence of quantum advantage.

### 3.9. Feature Importance

**Figure 11:**
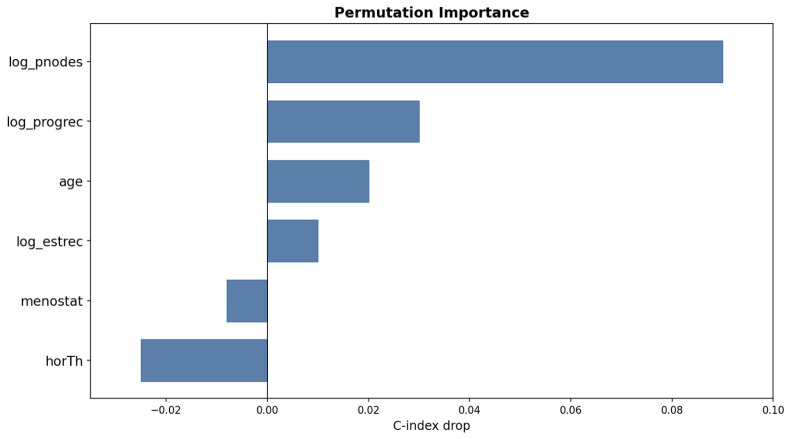
Permutation feature importance on the GBSG2 hold-out partition. Bars indicate the drop in concordance when the corresponding feature is independently shuffled.

Figure 11 reports permutation feature importance on the GBSG2 hold-out partition. The log-transformed number of positive lymph nodes dominated the model, with a concordance drop of approximately 0.09 when permuted. Progesterone receptor concentration followed with a drop of approximately 0.03, while age and oestrogen receptor concentration made smaller contributions. Menopausal status and hormonal therapy status showed near-zero or slightly negative importance, suggesting redundancy or noise-level contribution in the fitted model. This ordering is clinically plausible for node-positive breast cancer and confirms that the model recovered the same dominant signal emphasised by conventional prognostic tools.

## 4. Discussion

The principal finding is that a diagnostic-first, safety-gated quantum residual framework can distinguish between settings where quantum capacity is likely to be useful and settings where it should remain clinically inactive. On GBSG2, the leading classical and hybrid models achieved statistically comparable discrimination, and QResid-Boost tracked Cox-LASSO almost exactly. On FLChain, the hybrid model again preserved baseline performance without meaningful improvement. Conversely, on the synthetic high-frequency cohort, where the linear baseline performed near chance, QResid-Boost delivered its largest gain. This pattern supports a cautious but constructive interpretation: the framework does not establish broad quantum advantage for clinical survival analysis, but it does provide a reproducible mechanism for testing whether quantum enrichment is warranted.

For a high-impact clinical prediction journal, the most defensible contribution is therefore not a claim of universal quantum advantage. The stronger claim is methodological: quantum capacity can be embedded as an auditable, residual-only enrichment layer that is activated only when pre-training geometry and validation performance justify its use. This framing reduces the risk of technological overclaiming and positions the framework within the evidentiary standards expected for high-stakes AI models [8,9].

KTA-Survival is the methodological centre of this contribution. By adapting kernel-target alignment [20] to right-censored data, it offers a pre-training diagnostic that is both computationally inexpensive and directly tied to the survival-ranking objective. This is preferable to training a quantum model first and interpreting failure retrospectively. Nevertheless, KTA-Survival should not be read as a lower bound on eventual performance. It measures geometric compatibility, not optimisation success, calibration quality, or circuit sufficiency. The synthetic experiment makes this point clearly: alignment was positive, but the six-qubit circuit remained under-capacitated for the full periodic target.

QResid-Boost is clinically appealing because it constrains the role of the quantum component. The Cox-LASSO backbone provides interpretability and a stable baseline, while martingale-residual learning directs the VQC toward the unexplained component of risk. The α gate further prevents uncontrolled deviation from the classical model [9], an effect that is directly supported by the empirical behaviour of the gate. On GBSG2, where no exploitable residual signal remained, the learned gate stabilised at α ≈ 0.145 and the resulting test concordance was indistinguishable from Cox-LASSO (ΔC ≈ −0.0003; Section 3.4), so the bound demonstrably held the hybrid score next to the classical baseline. Conversely, on the synthetic high-frequency cohort, the gate opened to α ≈ 0.86 and the model added measurable discrimination (ΔC = +0.0397), confirming that the bound permits a controlled, data-driven correction precisely when residual structure is present. Because σ(αraw) is mathematically constrained to the interval (0, 1) (Section 2.5), the magnitude of the quantum contribution is bounded by construction and the two observed regimes (α near zero versus α approaching one) provide the analytical evidence requested for this claim. In practical terms, this means that quantum capacity is used as a residual enrichment layer, not as an opaque replacement for an established survival model. Such a design is aligned with the broader argument that high-stakes clinical prediction systems should prioritise transparency, auditability, and robust baseline comparisons [9].

The Quantum-Skip-Residual architecture extends this logic by importing the residual-learning principle into a hybrid quantum–classical setting [23]. The additive bypass forces the circuit to model deviation from a strong reference rather than the entire risk function. KTA-oriented pre-alignment and conservative parameter initialisation further reduce the instability associated with randomly initialised variational circuits [16,17]. These design choices do not eliminate the limitations of current quantum models, but they make the deployment question more disciplined: the quantum branch is only useful if the data contain residual structure that is both geometrically aligned and learnable within the available circuit budget.

The Stacking results reinforce this interpretation. Although heterogeneous ensembles often benefit from combining partially independent errors [27,39,40], the VQC component did not improve the GBSG2 stack. Removing it produced the same performance as RSF alone. The practical implication is straightforward: a quantum branch should be judged by its incremental contribution to a deployable pipeline, not by its novelty or standalone theoretical expressiveness. In this dataset, the quantum component was not sufficiently informative to justify inclusion in the final ensemble.

The WHAS500 external validation experiment highlights a separate issue: distribution shift. A model trained on breast-cancer recurrence did not transfer strongly to post-infarction mortality, regardless of modelling family. Classical ensembles retained moderate discrimination, whereas pure quantum learners approached chance. This is not a uniquely quantum failure; it reflects the broader challenge of transporting clinical prediction models across disease contexts, age distributions, endpoint definitions, and predictor meanings. Any clinical deployment would require local retraining, recalibration, and decision-analytic evaluation.

From a clinical perspective, the strongest internal GBSG2 models achieved discrimination that is competitive given the limited feature set. The analysis used only six routinely available clinicopathological variables, without genomic assays such as Oncotype DX [6] or Prosigna PAM50 [42]. This matters for settings where molecular profiling is unavailable or cost prohibitive. In such environments, a diagnostic-first enrichment layer may be useful if it can demonstrably improve risk stratification from standard pathology data while reporting α as an auditable indicator of how much the final score departs from the classical baseline.

Before clinical deployment, however, discrimination alone is insufficient. Decision curve analysis is needed to determine whether any model improves net benefit across clinically relevant threshold probabilities [35]. Given the small and non-significant differences among the leading GBSG2 models, it is plausible that decision curve analysis would show little or no net-benefit advantage for the hybrid model. Net reclassification improvement and integrated discrimination improvement could also be used to assess whether the Stacking ensemble or QResid-Boost meaningfully reclassifies patients compared with Cox-LASSO [10,38]. These analyses should be treated as mandatory in any translational extension of the present work.

The intended clinical workflow is therefore conservative. A standard pathological or Cox-based risk score would first be estimated. KTA-Survival would then be computed on the local institutional dataset to assess whether quantum enrichment is geometrically justified. Only if ΔKTA is positive and validation performance improves would QResid-Boost be deployed as an additive risk layer. In cardiovascular settings similar to WHAS500, local retraining is essential. The modular design is well suited to this process because the Cox-LASSO component can be refitted locally while the quantum residual branch can be warm-started and re-evaluated.

Regulatory and health-technology-assessment considerations further support this cautious workflow. AI-based clinical decision-support systems must demonstrate analytical validity, clinical validity, and clinical utility. The TRIPOD+AI guidance emphasises transparent reporting of model development, validation, intended use, and uncertainty [8]. The present study provides an analytical proof of concept and a reproducible diagnostic framework, but it does not establish prospective clinical utility. Before implementation as software as a medical device, the approach would require external validation in target populations, calibration assessment, decision curve analysis, and ideally a prospective impact study.

The NISQ findings are cautiously reassuring but should not be overstated. In this small six-qubit setting, depolarising and bit-flip noise did not materially alter concordance, likely because the risk ordering was preserved. This does not imply that larger, deeper, or hardware-executed circuits will behave similarly. It does, however, suggest that rank-based survival objectives may be relatively tolerant to certain forms of expectation-value attenuation, a property worth examining in future hardware-oriented studies.

This study has several limitations. First, all real datasets were low-dimensional, which restricts the range of possible quantum embedding effects. Higher-dimensional omics, radiomics, or multimodal clinical datasets may produce different results. Second, the synthetic positive control was intentionally designed to favour periodic encodings, so the observed gain should be interpreted as a regime demonstration rather than a general clinical effect. Third, KTA-Survival was benchmarked against a classical RBF reference; richer classical kernels could make the feasibility criterion more conservative [41]. Fourth, bootstrap intervals were stratified by event status but did not account fully for hyperparameter-selection uncertainty. Finally, clinical usefulness was not assessed with decision curve analysis, prospective validation, or workflow impact measures. A further limitation is that the current validation strategy does not fully quantify optimism introduced by model selection, hyperparameter tuning, and architecture selection. Although the held-out test set and bootstrap intervals provide useful uncertainty estimates, a nested resampling design or repeated external validation would provide a stronger estimate of generalisation. Subgroup performance, calibration slope/intercept, and decision-curve net benefit should also be reported before any clinical implementation claim is made.

## 5. Conclusions

This study introduces a diagnostic-first quantum residual boosting framework for clinical survival analysis. KTA-Survival provides a low-cost, pre-training test of whether a quantum embedding is geometrically aligned with a censored survival target. QResid-Boost then uses a Cox-LASSO backbone and a sigmoid-bounded quantum residual gate to ensure that the hybrid model remains anchored to an interpretable classical baseline. Across three real clinical cohorts and one synthetic positive control, the framework behaved as intended: it avoided harmful overcorrection when classical models already captured the dominant prognostic signal and produced its clearest gain when residual nonlinear periodic structure was present. These findings support a restrained but practical role for NISQ-era quantum components in clinical prediction: not as universal replacements for classical models, but as auditable, diagnostically screened enrichment layers within carefully validated survival-analysis pipelines. The principal, quantifiable outputs of this work are fourfold. First, KTA-Survival, a training-free feasibility diagnostic for censored data, yielded positive and statistically significant alignment on all evaluated cohorts (ΔKTA = +0.029, +0.024 and +0.018; permutation *p* = 0.003–0.031). Second, on low-dimensional clinical cohorts, the bounded gate kept the hybrid model statistically equivalent to Cox-LASSO (GBSG2 ΔC ≈ −0.0003; FLChain ΔC ≈ −0.0015), demonstrating the intended safety behaviour. Third, when genuine nonlinear periodic structure was present, the same framework delivered a measurable gain (synthetic ΔC = +0.0397). Fourth, the classical and hybrid models were statistically indistinguishable from one another yet significantly superior to every pure quantum variant (*p* ≤ 0.033), while the quantum residual’s probability estimates still required recalibration (integrated Brier score 0.192 versus 0.154 for Cox). Returning to the question posed in the title, the framework is best understood not as a claim that quantum residual boosting is uniformly beneficial, but as a disciplined answer to when it should be used: a diagnostic-first, gated quantum residual is justified only when a pre-training alignment screen is positive and validation performance improves, and it otherwise reduces safely to an interpretable classical survival model. Future work should extend the evaluation to higher-dimensional omics, radiomics, and multimodal cohorts in which nonlinear interactions are more plausible; replace the single held-out split with nested resampling or repeated external validation to quantify optimism from model and hyperparameter selection; add calibration (slope and intercept), decision-curve net benefit, and reclassification (NRI/IDI) analyses as prerequisites for any clinical-utility claim; benchmark KTA-Survival against richer classical kernels and examine deeper or wider circuits with explicit barren-plateau mitigation; and progress from noiseless and emulated-noise simulation toward execution on real NISQ hardware with error mitigation, ideally within a prospective impact study, before any deployment as software as a medical device.

## Figures and Tables

**Table 1:** Baseline characteristics of the principal cohorts.

Cohort	*n*	Age (Mean ± SD, Years)	Events (%)	Median Follow-Up (Days)
GBSG2 (Training)	686	53.1 ± 10.1	43.6	1084
WHAS500 (External validation)	500	69.8 ± 14.5	43.0	632

**Table 2:** Internal test performance on the GBSG2 hold-out partition.

Model	C-index	95% CI	NISQ	Source
Random Survival Forest	0.7188	[0.6864, 0.7508]	—	[2]
Stacking ensemble	0.7128	[0.6803, 0.7448]	—	This study
Cox-LASSO	0.7019	[0.6695, 0.7339]	—	[21]
QResid-Boost (Cox + α·VQC)	0.7016	[0.6692, 0.7336]	—	This study
VQC-Survival (sampled, 512 shots)	0.6191	[0.5786, 0.6592]	Partial	This study
VQC-Survival (NISQ)	0.5881	[0.5471, 0.6286]	Yes	This study
VQC-Survival (ideal)	0.5564	[0.5152, 0.5970]	—	This study
VQC-Ensemble (K = 3)	0.5062	[0.4644, 0.5481]	—	This study
DeepSurv	0.7035	[0.6709, 0.7355]	—	[3]

**Table 3:** Four-dimensional ablation along encoding, ansatz, ensemble size, and stacking configuration.

Dimension	Configuration	C-index
Encoding	Angle-Y (default)	0.4503
Encoding	Angle-Z	0.5627
Encoding	Angle-X	0.5293
Ansatz	Strongly Entangling (default)	0.5719
Ansatz	Basic Entangler	0.5508
Ansatz	Rotation-only (no entanglement)	0.4942
Ensemble size	K = 1	0.5494
Ensemble size	K = 2	0.5255
Ensemble size	K = 3 (default)	0.5062
Stacking	Full (VQC + RSF + Cox + DS)	0.7128
Stacking	VQC removed	0.7188
Stacking	VQC only	0.5062
Stacking	Equal weights	0.7128

**Table 4:** Zero-shot external validation on WHAS500. Bootstrap 95% confidence intervals based on 200 resamples. ΔC denotes the drop in concordance relative to the internal hold-out partition.

Model	C-index	95% CI	ΔC	Status
VQC-Survival	0.5254	[0.4828, 0.5640]	+0.031	OK
VQC-Ensemble (K = 3)	0.4943	[0.4547, 0.5333]	+0.012	OK
Stacking ensemble	0.6211	n/a	+0.092	Domain shift
Random Survival Forest (ref.)	0.6152	[0.5757, 0.6568]	+0.104	Domain shift

n/a: not available; CI: confidence interval.

**Table 5:** Multi-dataset evaluation with KTA-Survival diagnosis. ΔKTA is the alignment gain of the quantum kernel over the best-tuned classical RBF reference; ΔC is the test-concordance gain of QResid-Boost over Cox-LASSO.

Dataset	*n*	*p*	Events	Cox-LASSO	RSF	VQC	QResid	α	ΔC	ΔKTA	Verdict
GBSG2	686	6	43.6%	0.6486	0.7188	0.5402	0.6655	0.145	+0.0169	+0.0290	Positive
FLChain	1500	6	26.9%	0.8002	0.8118	0.5568	0.7988	0.96	−0.0015	+0.0241	Positive
Synthetic (HF-periodic)	600	6	40.8%	0.4857	0.6094	0.5050	0.5253	0.86	+0.0397	+0.0176	Positive

## Data Availability

All datasets used in this study (GBSG2, WHAS500, FLChain) are publicly available in the scikit-survival and lifelines libraries. The synthetic Weibull cohort is fully described by the data-generating process given and can be reproduced from the global random seed (42). The implementation will be made available on the corresponding author’s institutional repository upon acceptance.

## Supplementary Materials

The following supporting information can be downloaded at: https://www.mdpi.com/article/10.3390/jcm15145387/s1. Table S1. Pairwise paired-bootstrap comparison of the concordance index (C) among the four leading models on the GBSG2 held-out test set (*n* = 103, 45 events; B = 2000 bootstrap resamples). ΔC = C(A) − C(B); the 95% CI is the bootstrap percentile interval of ΔC and the *p*-value is two-sided. No comparison among the leading models reached significance (all *p* > 0.36), confirming that their concordance indices are statistically indistinguishable on this cohort. For reference, every leading model significantly exceeded both pure quantum variants (paired bootstrap *p* ≤ 0.033, falling to *p* ≈ 0.001 for the widest gaps).
