# Supplementary material for: Quantum Computing in a Diagnostic-First Quantum Residual Boosting Framework for Clinical Survival Analysis in Oncology and Cardiology
Source: J Clin Med. 2026 Jul 9;15(14):5387. doi: 10.3390/jcm15145387 (PMC13411548; doi:10.3390/jcm15145387)
Supplement: Supplementary file 1 [file jcm-15-05387-s001.zip › jcm-4338978-supplementary.pdf]

Table S1. Pairwise paired-bootstrap comparison of the concordance index (C) among the four leading models on the GBSG2 held-out test set (n = 103, 45 events; B = 2,000 bootstrap resamples). ΔC = C(A) − C(B); the 95% CI is the bootstrap percentile interval of ΔC and the p-value is two-sided. No comparison among the leading models reached significance (all $p > 0.36$), confirming that their concordance indices are statistically indistinguishable on this cohort. For reference, every leading model significantly exceeded both pure quantum variants (paired bootstrap $p \leq 0.033$, falling to $p \approx 0.001$ for the widest gaps).

**Comparison (A vs B)**	**C (A)**	**C (B)**	**ΔC**	**95% CI of ΔC**	**p-value**
RSF vs Stacking	0.7188	0.7128	+0.0060	[−0.023, +0.050]	0.556
RSF vs Cox-LASSO	0.7188	0.7019	+0.0169	[−0.026, +0.063]	0.415
RSF vs QResid-Boost	0.7188	0.7016	+0.0172	[−0.023, +0.067]	0.369
Stacking vs Cox-LASSO	0.7128	0.7019	+0.0109	[−0.021, +0.036]	0.686
Stacking vs QResid-Boost	0.7128	0.7016	+0.0112	[−0.020, +0.041]	0.587
Cox-LASSO vs QResid-Boost	0.7019	0.7016	+0.0003	[−0.010, +0.016]	0.712
